# Series of sums of products of higher-order Bernoulli functions

**DOI:** 10.1186/s13660-017-1494-9

**Published:** 2017-09-13

**Authors:** Taekyun Kim, Dae San Kim, Gwan-Woo Jang, Jongkyum Kwon

**Affiliations:** 1 Department of Mathematics, College of Science, Tianjin Polytechnic University, Tianjin, 300160 China; 2 Department of Mathematics, Kwangwoon University, Seoul, 139-701 Republic of Korea; 3 Department of Mathematics, Sogang University, Seoul, 121-742 Republic of Korea; 4 Department of Mathematics Education and RINS, Gyeongsang National University, Jinju, Gyeongsangnamdo 52828 Republic of Korea

**Keywords:** 11B68, 42A16, Fourier series, sums of products of higher-order Bernoulli functions

## Abstract

It is shown in a previous work that Faber-Pandharipande-Zagier’s and Miki’s identities can be derived from a polynomial identity, which in turn follows from the Fourier series expansion of sums of products of Bernoulli functions. Motivated by and generalizing this, we consider three types of functions given by sums of products of higher-order Bernoulli functions and derive their Fourier series expansions. Moreover, we express each of them in terms of Bernoulli functions.

## Introduction

Let *r* be a nonnegative integer. Then the Bernoulli polynomials $B_{n}^{(r)}(x)$ of order *r* are given by the generating function (see [1–7])

1.1 $$ \biggl(\frac{t}{e^{t}-1} \biggr)^{r} e^{xt} = \sum_{m=0} ^{\infty} B_{m}^{(r)}(x) \frac{t^{m}}{m!}. $$

When $x=0$, $B_{m}^{(r)} = B_{m}^{(r)}(0)$ are called the Bernoulli numbers of order *r*. In particular, $B_{m}(x) = B_{m}^{(1)}(x)$ are the ordinary Bernoulli polynomials.

As we can see from (1.1), the higher-order Bernoulli polynomials $B_{n}^{(r)}(x)$ are Appell polynomials and hence they satisfy

1.2 $$ \frac{d}{dx} B_{m}^{(r)}(x) = m B_{m-1}^{(r)}(x), \quad {m \geq1}. $$

Further, from (1.1), we can easily show that

1.3 $$ B_{m}^{(r)}(x+1) = B_{m}^{(r)}(x) + m B_{m-1}^{(r-1)}(x) \quad (m \geq1), $$

which in turn gives

1.4 $$ B_{m}^{(r)}(1) = B_{m}^{(r)} + m B_{m-1}^{(r-1)} \quad (m \geq1). $$

For any real number *x*, we let

$$ \langle x\rangle = x - [x] \in[0,1) $$

denote the fractional part of *x*. Then we recall here the following facts about the Fourier series expansion of the Bernoulli function $B_{n}(\langle x\rangle )$:

- for $m \geq2 $,
  1.5 $$ B_{m} \bigl(\langle x\rangle \bigr) = - m! \sum _{n= - \infty, n \neq0}^{\infty} \frac{e^{2\pi i nx}}{(2\pi i n)^{m}}, $$
- for $m = 1 $,
  1.6 $$ - \sum_{n= - \infty, n \neq0}^{\infty} \frac{e^{2\pi i nx}}{2\pi i n} = \textstyle\begin{cases} B_{1}(\langle x\rangle ), &\mbox{for } x\notin\mathbb{Z},\\ 0, &\mbox{for }x\in\mathbb{Z}. \end{cases} $$

In this paper, we will study the following three types of sums of products of higher-order Bernoulli functions and find Fourier series expansions for them. Moreover, we will express them in terms of Bernoulli functions. Let *r*, *s* be positive integers.

1. $\alpha_{m}(\langle x\rangle )=\sum_{k=0}^{m} B_{k}^{(r)}(\langle x\rangle ) B_{m-k}^{(s)}(\langle x\rangle )$ ($m \geq1$);
2. $\beta_{m}(\langle x\rangle )=\sum_{k=0}^{m}\frac{1}{k!(m-k)!} B_{k}^{(r)}(\langle x\rangle ) B_{m-k}^{(s)}(\langle x\rangle )$ ($m \geq1$);
3. $\gamma_{m}(\langle x\rangle )= \sum_{k=1}^{m-1}\frac{1}{k(m-k)} B_{k}^{(r)}(\langle x\rangle ) B_{m-k}^{(s)}(\langle x\rangle )$ ($m \geq2$).

For elementary facts about Fourier analysis, the reader may refer, for example, to [8–10].

As to $\gamma_{m}(\langle x\rangle )$, we note that the polynomial identity (1.7) follows immediately from the Fourier series expansion of $\gamma _{m}(\langle x\rangle )$ in Theorems 4.1 and 4.2:

1.7 $$\begin{aligned}& \sum_{k=1}^{m-1} \frac{1}{k(m-k)} B_{k}^{(r)}(x) B_{m-k}^{(s)}(x) \\& \quad =\frac{1}{m} \sum_{k=0}^{m} \binom{m}{k} \bigl\{ \Lambda_{m-k+1} + (H_{m-1}-H_{m-k}) \bigl(B_{m-k}^{(r-1)} + B_{m-k}^{(s-1)} \bigr) \bigr\} B_{k}(x), \end{aligned}$$

where, for each integer $l \geq2$,

1.8 $$ \Lambda_{l} = \sum _{k=1}^{l-1}\frac{1}{k(l-k)} \bigl((l-k)B_{k}^{(r)}B_{l-k-1}^{(s-1)} + k B_{k-1}^{(r-1)} B_{l-k}^{(s)} + k(l-k)B_{k-1}^{(r-1)}B_{l-k-1}^{(s-1)} \bigr), $$

and $H_{m}=\sum_{j=1}^{m}\frac{1}{j}$ are the harmonic numbers.

It is remarkable that the famous Faber-Pandharipande-Zagier identity (see [11, 12]) and the Miki identity (see [12–16]) can be easily derived from (1.7) and (1.8), with $r=s=1$. Below, we will give an outline for this and thus this may be viewed as our main motivation for the present study.

Indeed, from (1.7) and (1.8), with $r=s=1$, we get

1.9 $$\begin{aligned}& \sum_{k=1}^{m-1} \frac{1}{k (m-k )}B_{k} (x )B_{m-k} (x ) \\& \quad = \frac{2}{m^{2}} \biggl(B_{m}+\frac{1}{2} \biggr)+ \frac{2}{m}\sum_{k=1}^{m-2} \frac{1}{m-k}\binom{m}{k}B_{m-k}B_{k} (x )+ \frac{2}{m}H_{m-1}B_{m} (x ) \quad(m\geq2). \end{aligned}$$

Simple modification of (1.9) yields

1.10 $$\begin{aligned}& \sum_{k=1}^{m-1} \frac{1}{2k (2m-2k )}B_{2k} (x )B_{2m-2k} (x )+ \frac{2}{2m-1}B_{1} (x )B_{2m-1} (x ) \\& \quad = \frac{1}{m}\sum_{k=1}^{m} \frac{1}{2k}\binom {2m}{2k}B_{2k}B_{2m-2k} (x )+ \frac{1}{m}H_{2m-1}B_{2m} (x ) \\& \qquad{} +\frac{2}{2m-1}B_{1} (x )B_{2m-1}\quad (m\ge2 ). \end{aligned}$$

Letting $x=0$ in (1.10) gives a slightly different version of the well-known Miki identity (see [15]):

1.11 $$\begin{aligned}& \sum_{k=1}^{m-1} \frac{1}{2k (2m-2k )}B_{2k}B_{2m-2k} \\& \quad = \frac{1}{m}\sum_{k=1}^{m} \frac{1}{2k}\binom {2m}{2k}B_{2k}B_{2m-2k}+ \frac{1}{m}H_{2m-1}B_{2m}\quad (m\ge2 ). \end{aligned}$$

Setting $x=\frac{1}{2}$ in (1.11) with $\overline{B}_{m}= (\frac{1-2^{m-1}}{2^{m-1}} )B_{m}= (2^{1-m}-1 )B_{m}=B_{m} (\frac{1}{2} )$, we have

1.12 $$\begin{aligned}& \sum_{k=1}^{m-1} \frac{1}{2k (2m-2k )}\overline {B}_{2k}\overline{B}_{2m-2k} \\& \quad = \frac{1}{m}\sum_{k=1}^{m} \frac{1}{2k}\binom {2m}{2k}B_{2k}\overline{B}_{2m-2k}+ \frac{1}{m}H_{2m-1}\overline {B}_{2m}\quad (m\ge2 ), \end{aligned}$$

which is the Faber-Pandharipande-Zagier identity (see [11]). Some of the different proofs of Miki’s identity can be found in [13–16]. Miki in [15] exploits a formula for the Fermat quotient $\frac{a^{p}-a}{p}$ modulo $p^{2}$, Shiratani-Yokoyama in [16] employs *p*-adic analysis, Gessel in [14] is based on two different expressions for Stirling numbers of the second kind $S_{2} (n,k )$, and Dunne-Schubert in [13] uses the asymptotic expansion of some special polynomials coming from the quantum field theory computations As we can see, all of these proofs are quite involved. On the other hand, our proofs of Miki’s and Faber-Pandharipande-Zagier’s identities follow from the polynomial identity (1.9), which in turn follows immediately the Fourier series expansion of $\gamma_{m}(\langle x\rangle )$ in Theorems 4.1 and 4.2, with $r=s=1$, together with the elementary manipulations outlined in (1.9)-(1.12). Some related recent work can be found in [17–21].

## The function $\alpha_{m}(\langle x\rangle )$

Let $\alpha_{m} (x) = \sum_{k=0}^{m} B_{k}^{(r)}(x) B_{m-k}^{(s)}(x)$ ($m \geq1$). Then we will consider the function

$$ \alpha_{m} \bigl(\langle x\rangle \bigr)=\sum _{k=0}^{m} B_{k}^{(r)} \bigl(\langle x\rangle \bigr) B_{m-k}^{(s)} \bigl(\langle x\rangle \bigr)\quad (m \geq1), $$

defined on $\mathbb{R}$, which is periodic with period 1.

The Fourier series of $\alpha_{m} (\langle x\rangle )$ is

2.1 $$ \sum_{n= -\infty}^{\infty} A_{n}^{(m)} e^{2\pi i nx}, $$

where

2.2 $$ A_{n}^{(m)}= \int_{0} ^{1} \alpha_{m} \bigl(\langle x \rangle \bigr) e^{-2\pi i nx} \,dx = \int_{0} ^{1} \alpha_{m} (x) e^{-2\pi i nx} \,dx. $$

To continue our discussion, we need to observe the following:

2.3 $$ \begin{aligned}[b] \alpha_{m}'(x)& = \sum_{k=0}^{m} \bigl(k B_{k-1}^{(r)}(x) B_{m-k}^{(s)}(x) + (m-k)B_{k}^{(r)}(x) B_{m-k-1}^{(s)}(x) \bigr) \\ & = \sum_{k=1}^{m} k B_{k-1}^{(r)}(x) B_{m-k}^{(s)}(x) + \sum_{k=0}^{m-1}(m-k)B_{k}^{(r)}(x) B_{m-k-1}^{(s)}(x) \\ & = \sum_{k=0}^{m-1} (k+1) B_{k}^{(r)}(x) B_{m-1-k}^{(s)}(x) + \sum _{k=0}^{m-1}(m-k)B_{k}^{(r)}(x) B_{m-1-k}^{(s)}(x) \\ & = (m+1) \sum_{k=0}^{m-1} B_{k}^{(r)}(x) B_{m-1-k}^{(s)}(x) = (m+1) \alpha_{m-1}(x). \end{aligned} $$

From this, we obtain

2.4 $$ \biggl(\frac{\alpha_{m+1}(x)}{m+2} \biggr)' = \alpha_{m}(x) $$

and

2.5 $$ \int_{0} ^{1} \alpha_{m}(x) \,dx = \frac{1}{m+2} \bigl(\alpha_{m+1}(1) - \alpha_{m+1} (0) \bigr). $$

For $m \geq1 $, we set

2.6 $$\begin{aligned} \Delta_{m} =& \alpha_{m}(1) - \alpha_{m} (0) = \sum_{k=0}^{m} \bigl(B_{k}^{(r)}(1)B_{m-k}^{(s)}(1) - B_{k}^{(r)}B_{m-k}^{(s)} \bigr) \\ =& \sum_{k=0}^{m} \bigl( \bigl(B_{k}^{(r)}+ k B_{k-1}^{(r-1)} \bigr) \bigl(B_{m-k}^{(s)}+ (m-k)B_{m-k-1}^{(s-1)} \bigr) - B_{k}^{(r)}B_{m-k}^{(s)} \bigr) \\ =& \sum_{k=0}^{m} \bigl((m-k)B_{k}^{(r)}B_{m-k-1}^{(s-1)} + k B_{k-1}^{(r-1)} B_{m-k}^{(s)} + k(m-k)B_{k-1}^{(r-1)}B_{m-k-1}^{(s-1)} \bigr). \end{aligned}$$

Now, we have

2.7 $$ \alpha_{m}(1) = \alpha_{m}(0) \quad\Longleftrightarrow\quad \Delta_{m} = 0 $$

and

2.8 $$ \int_{0} ^{1} \alpha_{m}(x) \,dx = \frac{1}{m+2} \Delta_{m+1}. $$

We now would like to determine the Fourier coefficients $A_{n}^{(m)}$.

*Case* 1: $n \neq0 $.

2.9 $$ \begin{aligned}[b] A_{n}^{(m)} & = \int_{0} ^{1} \alpha_{m} (x) e^{-2\pi i nx} \,dx \\ & = - \frac{1}{2\pi i n} \bigl[\alpha_{m} (x) e^{-2\pi i nx} \bigr]_{0}^{1} + \frac {1}{2\pi i n} \int_{0} ^{1} \alpha_{m}' (x) e^{-2\pi i nx} \,dx \\ & = - \frac{1}{2\pi i n} \bigl(\alpha_{m} (1) - \alpha_{m} (0) \bigr) + \frac{m+1}{2\pi i n} \int_{0} ^{1} \alpha_{m-1}(x) e^{-2\pi i nx} \,dx \\ & = \frac{m+1}{2\pi i n} A_{n}^{(m-1)} - \frac{1}{2 \pi i n} \Delta_{m}, \end{aligned} $$

from which by induction on *m*, we can easily derive that

2.10 $$ A_{n}^{(m)} = - \frac{1}{m+2} \sum _{j=1}^{m} \frac{(m+2)_{j}}{(2\pi i n)^{j}} \Delta_{m-j+1}. $$

*Case* 2: $n = 0$.

2.11 $$ A_{0}^{(m)} = \int_{0} ^{1} \alpha_{m} (x) \,dx = \frac{1}{m+2}\Delta_{m+1}. $$

$\alpha_{m}(\langle x\rangle )$ ($m \geq1$) is piecewise $C^{\infty}$. In addition, $\alpha_{m}(\langle x\rangle ) $ is continuous for those positive integers *m* with $\Delta_{m} = 0$, and discontinuous with jump discontinuities at integers for those positive integers with $\Delta_{m} \neq0$.

Assume first that $\Delta_{m} = 0$, for a positive integer *m*. Then $\alpha_{m}(0) = \alpha_{m}(1)$. Hence $\alpha_{m} (\langle x\rangle )$ is piecewise $C^{\infty}$ and continuous. Thus the Fourier series of $\alpha_{m} (\langle x\rangle )$ converges uniformly to $\alpha_{m} (\langle x\rangle )$, and

2.12 $$ \begin{aligned}[b] \alpha_{m} \bigl(\langle x \rangle \bigr) ={}& \frac{1}{m+2}\Delta_{m+1} + \sum _{n= - \infty, n \neq0}^{\infty} \Biggl(-\frac{1}{m+2}\sum _{j=1}^{m}\frac{(m+2)_{j}}{(2\pi i n)^{j}} \Delta_{m-j+1} \Biggr)e^{2\pi i nx} \\ ={}& \frac{1}{m+2} \Delta_{m+1} + \frac{1}{m+2} \sum _{j=1}^{m} \binom{m+2}{j} \Delta_{m-j+1} \Biggl( -j! \sum_{n= - \infty, n \neq 0}^{\infty} \frac{e^{2\pi i nx}}{(2\pi i n)^{j}} \Biggr) \\ ={}& \frac{1}{m+2} \Delta_{m+1} + \frac{1}{m+2} \sum _{j=2}^{m} \binom{m+2}{j} \Delta_{m-j+1} B_{j} \bigl(\langle x\rangle \bigr) \\ &{} + \Delta_{m} \times \textstyle\begin{cases} B_{1}(\langle x\rangle ), &\mbox{for } x \notin\mathbb{Z},\\ 0,&\mbox{for }x \in\mathbb{Z}. \end{cases}\displaystyle \end{aligned} $$

We are now ready to state our first result.

### Theorem 2.1

*For each positive integer* *l*, *let*

$$\Delta_{l} = \sum_{k=0}^{l} \bigl((l-k)B_{k}^{(r)}B_{l-k-1}^{(s-1)} + k B_{k-1}^{(r-1)} B_{l-k}^{(s)} + k(l-k)B_{k-1}^{(r-1)}B_{l-k-1}^{(s-1)} \bigr). $$

*Assume that* $\Delta_{m} = 0$, *for a positive integer* *m*. *Then we have the following*.

(a) $\sum_{k=0}^{m} B_{k}^{(r)}(\langle x\rangle ) B_{m-k}^{(s)}(\langle x\rangle )$ *has the Fourier series expansion*

2.13 $$ \begin{aligned}[b] & \sum_{k=0}^{m} B_{k}^{(r)} \bigl(\langle x\rangle \bigr) B_{m-k}^{(s)} \bigl(\langle x\rangle \bigr) \\ &\quad = \frac{1}{m+2} \Delta_{m+1} + \sum _{n= - \infty, n \neq 0}^{\infty} \Biggl(-\frac{1}{m+2} \sum _{j=1}^{m} \frac{(m+2)_{j}}{(2\pi i n)^{j}}\Delta_{m-j+1} \Biggr)e^{2\pi i nx}, \end{aligned} $$

*for all* $x \in \mathbb{R}$, *where the convergence is uniform*.

2.14 $$ \mathrm{(b)}\quad \sum_{k=0}^{m} B_{k}^{(r)} \bigl(\langle x\rangle \bigr) B_{m-k}^{(s)} \bigl(\langle x \rangle \bigr) = \frac{1}{m+2} \sum_{j=0,j \neq1}^{m} \binom{m+2}{j} \Delta_{m-j+1} B_{j} \bigl(\langle x\rangle \bigr), $$

*for all* *x* *in* $\mathbb{R}$.

Assume next that $\Delta_{m} \neq0$, for a positive integer *m*. Then $\alpha_{m} (0) \neq\alpha_{m} (1)$. Hence $\alpha_{m} (\langle x\rangle )$ is piecewise $C^{\infty}$, and discontinuous with jump discontinuities at integers.

The Fourier series of $\alpha_{m} (\langle x\rangle )$ converges pointwise to $\alpha _{m} (\langle x\rangle )$, for $x \notin\mathbb{Z}$, and converges to

2.15 $$ \frac{1}{2} \bigl(\alpha_{m} (0)+ \alpha_{m} (1) \bigr) = \alpha_{m} (0) + \frac{1}{2} \Delta_{m}, $$

for $x \in\mathbb{Z}$.

Now, we are ready to state our second result.

### Theorem 2.2

*For each positive integer* *l*, *let*

$$\Delta_{l} = \sum_{k=0}^{l} \bigl((l-k)B_{k}^{(r)}B_{l-k-1}^{(s-1)} + k B_{k-1}^{(r-1)} B_{l-k}^{(s)} + k(l-k)B_{k-1}^{(r-1)}B_{l-k-1}^{(s-1)} \bigr). $$

*Assume that* $\Delta_{m} \neq0$, *for a positive integer* *m*. *Then we have the following*.

2.16 $$\begin{aligned} &\begin{aligned}[b] &\mathrm{(a)\quad} \frac{1}{m+2} \Delta_{m+1} + \sum _{n= - \infty, n \neq 0}^{\infty} \Biggl(- \frac{1}{m+2} \sum _{j=1}^{m} \frac {(m+2)_{j}}{(2\pi i n)^{j}} \Delta_{m-j+1} \Biggr)e^{2\pi i nx} \\ &\phantom{\mathrm{(a)\qquad}} = \textstyle\begin{cases} \sum_{k=0}^{m} B_{k}^{(r)}(\langle x\rangle ) B_{m-k}^{(s)}(\langle x\rangle ), &\textit{for } x \notin\mathbb{Z},\\ \sum_{k=0}^{m} B_{k}^{(r)}B_{m-k}^{(s)} + \frac{1}{2} \Delta_{m}, &\textit{for } x \in\mathbb{Z}. \end{cases}\displaystyle \end{aligned} \end{aligned}$$

2.17 $$\begin{aligned} &\begin{aligned}[b] &\mathrm{(b)\quad} \frac{1}{m+2} \sum_{j=0}^{m} \binom{m+2}{j} \Delta_{m-j+1} B_{j} \bigl(\langle x\rangle \bigr) \\ &\phantom{\mathrm{(b)\qquad}} = \sum_{k=0}^{m} B_{k}^{(r)} \bigl(\langle x\rangle \bigr) B_{m-k}^{(s)} \bigl(\langle x \rangle \bigr), \quad\textit{for } x \notin \mathbb{Z}; \end{aligned} \end{aligned}$$

2.18 $$\begin{aligned} &\begin{aligned}[b] &\phantom{\mathrm{(b)\quad}} \frac{1}{m+2} \sum_{j=0,j \neq1}^{m} \binom{m+2}{j} \Delta_{m-j+1} B_{j} \bigl(\langle x\rangle \bigr) \\ &\phantom{\mathrm{(b)\qquad}} = \sum_{k=0}^{m} B_{k}^{(r)}B_{m-k}^{(s)} + \frac{1}{2} \Delta_{m}, \quad\textit{for } x \in\mathbb{Z}. \end{aligned} \end{aligned}$$

## The function $\beta_{m}(\langle x\rangle )$

Let $\beta_{m}(x)=\sum_{k=0}^{m} \frac{1}{k!(m-k)!} B_{k}^{(r)}(x) B_{m-k}^{(s)}(x)$ ($m \geq1$). Then we will study the function

$$\beta_{m} \bigl(\langle x\rangle \bigr) = \sum _{k=0}^{m} \frac{1}{k!(m-k)!} B_{k}^{(r)} \bigl(\langle x\rangle \bigr) B_{m-k}^{(s)} \bigl(\langle x \rangle \bigr) \quad (m \geq1), $$

defined on $\mathbb{R}$, which is periodic with period 1.

The Fourier series of $\beta_{m}(\langle x\rangle )$ is

3.1 $$ \sum_{n=-\infty}^{\infty}B_{n}^{(m)}e^{2\pi i n x}, $$

where

3.2 $$ B_{n}^{(m)}= \int_{0}^{1}\beta_{m} \bigl(\langle x \rangle \bigr)e^{-2\pi i n x}\,dx= \int _{0}^{1}\beta_{m}(x)e^{-2\pi i n x}\,dx. $$

Before proceeding, we need to observe the following:

3.3 $$ \begin{aligned}[b] \beta'_{m}(x)={}& \sum_{k=0}^{m} \biggl\{ \frac {k}{k!(m-k)!}B_{k-1}^{(r)}(x) B_{m-k}^{(s)}(x) +\frac{(m-k)}{k!(m-k)!}B_{k}^{(r)}(x) B_{m-k-1}^{(s)}(x) \biggr\} \\ ={}&\sum_{k=1}^{m}\frac{1}{(k-1)!(m-k)!}B_{k-1}^{(r)}(x) B_{m-k}^{(s)}(x) \\ &{} +\sum_{k=0}^{m-1}\frac{1}{k!(m-k-1)!}B_{k}^{(r)}(x) B_{m-k-1}^{(s)}(x) \\ ={}&\sum_{k=0}^{m-1}\frac{1}{k!(m-1-k)!}B_{k}^{(r)}(x) B_{m-1-k}^{(s)}(x) \\ &{} +\sum_{k=0}^{m-1}\frac{1}{k!(m-1-k)!}B_{k}^{(r)}(x) B_{m-1-k}^{(s)}(x) \\ ={}& 2\beta_{m-1}(x). \end{aligned} $$

From this, we get

3.4 $$ \biggl(\frac{\beta_{m+1}(x)}{2} \biggr)' = \beta_{m}(x) $$

and

3.5 $$ \int_{0}^{1}\beta_{m}(x)\,dx= \frac{1}{2} \bigl(\beta_{m+1}(1)-\beta _{m+1}(0) \bigr). $$

For $m \geq1 $, we put

3.6 $$ \begin{aligned}[b] \Omega_{m} ={}& \beta_{m}(1) - \beta_{m}(0) \\ ={}& \sum_{k=0}^{m} \frac{1}{k!(m-k)!} \bigl(B_{k}^{(r)}(1) B_{m-k}^{(s)}(1) - B_{k}^{(r)} B_{m-k}^{(s)} \bigr) \\ ={}& \sum_{k=0}^{m} \frac{1}{k!(m-k)!} \bigl( \bigl(B_{k}^{(r)}+ k B_{k-1}^{(r-1)} \bigr) \bigl(B_{m-k}^{(s)}+ (m-k)B_{m-k-1}^{(s-1)} \bigr) - B_{k}^{(r)}B_{m-k}^{(s)} \bigr) \\ ={}& \sum_{k=0}^{m}\frac{1}{k!(m-k)!} \bigl((m-k)B_{k}^{(r)}B_{m-k-1}^{(s-1)} + k B_{k-1}^{(r-1)} B_{m-k}^{(s)} \\ &{} + k(m-k)B_{k-1}^{(r-1)}B_{m-k-1}^{(s-1)} \bigr). \end{aligned} $$

Now

3.7 $$ \beta_{m}(0) = \beta_{m}(1) \quad\Longleftrightarrow\quad \Omega_{m} = 0 $$

and

3.8 $$ \int_{0}^{1}\beta_{m}(x)\,dx = \frac{1}{2}\Omega_{m+1}. $$

We now want to determine the Fourier coefficients $B_{n}^{(m)}$.

*Case* 1: $n\neq0$

3.9 $$ \begin{aligned}[b] B_{n}^{(m)} & = \int_{0}^{1}\beta_{m}(x)e^{-2\pi i n x}\,dx \\ & = - \frac{1}{2\pi i n} \bigl[\beta_{m}(x)e^{-2\pi i n x} \bigr]_{0}^{1} + \frac{1}{2\pi i n} \int_{0}^{1}\beta'_{m}(x)e^{-2\pi i n x}\,dx \\ & = - \frac{1}{2\pi i n} \bigl(\beta_{m}(1)-\beta_{m}(0) \bigr) + \frac{2}{2\pi i n} \int_{0}^{1}\beta_{m-1}(x)e^{-2\pi i n x}\,dx \\ & = \frac{2}{2\pi i n}B_{n}^{(m-1)} - \frac{1}{2\pi i n} \Omega_{m}. \end{aligned} $$

From this, we easily get the following result by induction on *m*:

3.10 $$ B_{n}^{(m)} = - \sum _{j=1}^{m} \frac{2^{j-1}}{(2\pi i n)^{j}}\Omega_{m-j+1}. $$

*Case* 2: $n=0$

3.11 $$ B_{0}^{(m)} = \int_{0}^{1}\beta_{m}(x)\,dx= \frac{1}{2}\Omega_{m+1}. $$

$\beta_{m}(\langle x\rangle )$ ($m \geq1$) is piecewise $C^{\infty}$. Moreover, $\beta_{m}(\langle x\rangle )$ is continuous for those positive integers *m* with $\Omega_{m} = 0$ and discontinuous with jump discontinuities at integers for those positive integers *m* with $\Omega_{m} \neq0$.

Assume first that $\Omega_{m} = 0$, for a positive integer *m*. Then $\beta_{m}(0) = \beta_{m}(1)$. Hence $\beta_{m}(\langle x\rangle )$ is piecewise $C^{\infty}$ and continuous. Thus the Fourier series of $\beta_{m}(\langle x\rangle )$ converges uniformly to $\beta_{m}(\langle x\rangle )$, and

3.12 $$\begin{aligned} \beta_{m} \bigl(\langle x \rangle \bigr) =& \frac{1}{2} \Omega_{m+1} + \sum_{n=-\infty,n\neq0}^{\infty} \Biggl(-\sum_{j=1}^{m}\frac{2^{j-1}}{(2\pi i n)^{j}} \Omega _{m-j+1} \Biggr)e^{2\pi i n x} \\ =& \frac{1}{2} \Omega_{m+1} + \sum_{j=1}^{m} \frac{2^{j-1}}{j!} \Omega_{m-j+1} \Biggl(-j!\sum _{n=-\infty,n\neq0}^{\infty}\frac {e^{2\pi n x}}{(2\pi i n)^{j}} \Biggr) \\ =&\frac{1}{2} \Omega_{m+1} + \sum_{j=2}^{m} \frac{2^{j-1}}{j!} \Omega_{m-j+1}B_{j} \bigl(\langle x\rangle \bigr) + \Omega_{m} \times \textstyle\begin{cases} B_{1}(\langle x\rangle ),&\mbox{for } x\notin\mathbb{Z},\\ 0,&\mbox{for } x\in\mathbb{Z}. \end{cases}\displaystyle \end{aligned}$$

Now, we can state our first result.

### Theorem 3.1

*For each positive integer* *l*, *let*

3.13 $$ \Omega_{l} = \sum _{k=0}^{l}\frac{1}{k!(l-k)!} \bigl((l-k)B_{k}^{(r)}B_{l-k-1}^{(s-1)} + k B_{k-1}^{(r-1)} B_{l-k}^{(s)} + k(l-k)B_{k-1}^{(r-1)}B_{l-k-1}^{(s-1)} \bigr). $$

*Assume that* $\Omega_{m} = 0$, *for a positive integer* *m*. *Then we have the following*.

(a) $\sum_{k=0}^{m}\frac{1}{k!(m-k)!}B_{k}^{(r)}(\langle x\rangle ) B_{m-k}^{(s)}(\langle x\rangle )$ *has the Fourier expansion*

3.14 $$ \begin{aligned}[b] & \sum_{k=0}^{m} \frac{1}{k!(m-k)!} B_{k}^{(r)} \bigl(\langle x\rangle \bigr) B_{m-k}^{(s)} \bigl(\langle x\rangle \bigr) \\ &\quad =\frac{1}{2} \Omega_{m+1} + \sum_{n=-\infty,n\neq0}^{\infty} \Biggl(-\sum_{j=1}^{m}\frac{2^{j-1}}{(2\pi i n)^{j}} \Omega _{m-j+1} \Biggr)e^{2\pi i n x}, \end{aligned} $$

*for all* $x \in\mathbb{R}$, *where the convergence is uniform*.

3.15 $$ \mathrm{(b)}\quad \sum_{k=0}^{m} \frac{1}{k!(m-k)!} B_{k}^{(r)} \bigl(\langle x\rangle \bigr) B_{m-k}^{(s)} \bigl(\langle x\rangle \bigr) = \sum_{j=0,j\neq1}^{m}\frac{2^{j-1}}{j!} \Omega_{m-j+1}B_{j} \bigl(\langle x\rangle \bigr), $$

*for all* $x \in\mathbb{R}$.

Assume next that $\Omega_{m} \neq0$, for a positive integer *m*. Then $\beta_{m}(0) \neq\beta_{m}(1)$. Hence $\beta_{m}(\langle x\rangle )$ is piecewise $C^{\infty}$, and discontinuous with jump discontinuities at integers. Then the Fourier series of $\beta_{m}(\langle x\rangle )$ converges pointwise to $\beta_{m}(\langle x\rangle )$, for $x\notin\mathbb{Z}$, and converges to

3.16 $$ \frac{1}{2} \bigl(\beta_{m}(0)+ \beta_{m}(1) \bigr)=\beta_{m}(0) + \frac{1}{2} \Omega_{m}, $$

for $x\in\mathbb{Z}$.

Now, we can state our second result.

### Theorem 3.2

*For each positive integer* *l*, *let*

3.17 $$ \Omega_{l} = \sum _{k=0}^{l}\frac{1}{k!(l-k)!} \bigl((l-k)B_{k}^{(r)}B_{l-k-1}^{(s-1)} + k B_{k-1}^{(r-1)} B_{l-k}^{(s)} + k(l-k)B_{k-1}^{(r-1)}B_{l-k-1}^{(s-1)} \bigr). $$

*Assume that* $\Omega_{m} \neq0$, *for a positive integer* *m*. *Then we have the following*:

3.18 $$\begin{aligned} &\begin{aligned}[b] & \mathrm{(a)}\quad \frac{1}{2} \Omega_{m+1} + \sum_{n=-\infty,n\neq0}^{\infty} \Biggl(-\sum_{j=1}^{m}\frac{2^{j-1}}{(2\pi i n)^{j}} \Omega _{m-j+1} \Biggr)e^{2\pi i n x} \\ &\phantom{\mathrm{(a)}\quad}\quad = \textstyle\begin{cases} \sum_{k=0}^{m}\frac{1}{k!(m-k)!}B_{k}^{(r)}(\langle x\rangle ) B_{m-k}^{(s)}(\langle x\rangle ), &\textit{for } x\notin\mathbb{Z}, \\ \sum_{k=0}^{m}\frac{1}{k!(m-k)!}B_{k}^{(r)} B_{m-k}^{(s)} + \frac {1}{2} \Omega_{m}, &\textit{for } x\in\mathbb{Z}. \end{cases}\displaystyle \end{aligned} \end{aligned}$$

3.19 $$\begin{aligned} & \begin{aligned}[b] &\mathrm{(b)}\quad \sum _{j=0}^{m}\frac{2^{j-1}}{j!} \Omega_{m-j+1}B_{j} \bigl(\langle x\rangle \bigr) \\ &\phantom{\mathrm{(b)}\quad}\quad = \sum_{k=0}^{m}\frac{1}{k!(m-k)!}B_{k}^{(r)} \bigl(\langle x\rangle \bigr) B_{m-k}^{(s)} \bigl(\langle x \rangle \bigr), \quad\textit{for } x \notin\mathbb{Z}; \sum_{j=0,j \neq1}^{m}\frac{2^{j-1}}{j!} \Omega _{m-j+1}B_{j} \bigl(\langle x\rangle \bigr) \\ &\phantom{\mathrm{(b)}\quad}\quad = \sum_{k=0}^{m}\frac{1}{k!(m-k)!}B_{k}^{(r)} B_{m-k}^{(s)} + \frac {1}{2} \Omega_{m},\quad \textit{for } x\in\mathbb{Z}. \end{aligned} \end{aligned}$$

## The function $\gamma_{m}(\langle x\rangle )$

Let $\gamma_{m}(x)=\sum_{k=1}^{m-1}\frac{1}{k(m-k)} B_{k}^{(r)}(x) B_{m-k}^{(s)}(x)$ ($m \geq2$). Then we will investigate the function

$$\gamma_{m} \bigl(\langle x\rangle \bigr)=\sum _{k=1}^{m-1}\frac{1}{k(m-k)}B_{k}^{(r)} \bigl(\langle x\rangle \bigr) B_{m-k}^{(s)} \bigl(\langle x \rangle \bigr), $$

defined on $\mathbb{R}$, which is periodic with period 1.

The Fourier series of $\gamma_{m}(\langle x\rangle )$ is

4.1 $$ \sum_{n=-\infty}^{\infty}C_{n}^{(m)}e^{2\pi i n x}, $$

where

4.2 $$ C_{n}^{(m)}= \int_{0}^{1}\gamma_{m} \bigl(\langle x \rangle \bigr)e^{-2\pi i n x}\,dx= \int _{0}^{1}\gamma_{m}(x)e^{-2\pi i n x}\,dx. $$

To proceed, we need to observe the following:

4.3 $$\begin{aligned} \gamma_{m}'(x) =& \sum_{k=1}^{m-1}\frac{1}{m-k}B_{k-1}^{(r)}(x) B_{m-k}^{(s)}(x) + \sum_{k=1}^{m-1} \frac{1}{k}B_{k}^{(r)}(x) B_{m-k-1}^{(s)}(x) \\ =&\sum_{k=0}^{m-2} \frac{1}{m-1-k} B_{k}^{(r)}(x) B_{m-1-k}^{(s)}(x) + \sum _{k=1}^{m-1} \frac{1}{k} B_{k}^{(r)}(x) B_{m-1-k}^{(s)}(x) \\ =&\sum_{k=1}^{m-2} \biggl( \frac{1}{m-1-k}+\frac{1}{k} \biggr) B_{k}^{(r)}(x) B_{m-1-k}^{(s)}(x) + \frac{1}{m-1} B_{m-1}^{(s)}(x) + \frac{1}{m-1} B_{m-1}^{(r)}(x) \\ =& (m-1)\sum_{k=1}^{m-2} \frac{1}{k(m-1-k)} B_{k}^{(r)}(x) B_{m-1-k}^{(s)}(x) + \frac{1}{m-1} B_{m-1}^{(s)}(x) + \frac{1}{m-1} B_{m-1}^{(r)}(x) \\ =& (m-1)\gamma_{m-1}(x) + \frac{1}{m-1} B_{m-1}^{(s)}(x) + \frac {1}{m-1} B_{m-1}^{(r)}(x). \end{aligned}$$

From this, we easily obtain

4.4 $$ \gamma_{m}(x)= \biggl(\frac{1}{m} \biggl( \gamma_{m+1}(x)-\frac {1}{m(m+1)} B_{m+1}^{(r)}(x) - \frac{1}{m(m+1)} B_{m+1}^{(s)}(x) \biggr) \biggr)' $$

and

4.5 $$\begin{aligned} \int_{0}^{1}\gamma_{m}(x)\,dx =&\frac{1}{m} \biggl[\gamma_{m+1}(x)-\frac{1}{m(m+1)} B_{m+1}^{(r)}(x) - \frac{1}{m(m+1)} B_{m+1}^{(s)}(x) \biggr]_{0}^{1} \\ =& \frac{1}{m} \biggl(\gamma_{m+1}(1)-\gamma_{m+1}(0) - \frac{1}{m(m+1)} \bigl(B_{m+1}^{(r)}(1) - B_{m+1}^{(r)}(0) \bigr) \\ &{} - \frac{1}{m(m+1)} \bigl(B_{m+1}^{(s)}(1)- B_{m+1}^{(s)}(0) \bigr) \biggr) \\ =& \frac{1}{m} \biggl(\gamma_{m+1}(1)-\gamma_{m+1}(0)- \frac {1}{m}B_{m}^{(r-1)} - \frac{1}{m}B_{m}^{(s-1)} \biggr). \end{aligned}$$

Let $\Lambda_{1} = 0$, and for $m \geq2$, we let

4.6 $$\begin{aligned} \Lambda_{m} =& \gamma_{m}(1)-\gamma_{m}(0) \\ =&\sum_{k=1}^{m-1}\frac{1}{k(m-k)} \bigl(B_{k}^{(r)}(1) B_{m-k}^{(s)}(1) - B_{k}^{(r)} B_{m-k}^{(s)} \bigr) \\ =&\sum_{k=1}^{m-1}\frac{1}{k(m-k)} \bigl( \bigl(B_{k}^{(r)}+ k B_{k-1}^{(r-1)} \bigr) \bigl(B_{m-k}^{(s)}+ (m-k)B_{m-k-1}^{(s-1)} \bigr) - B_{k}^{(r)}B_{m-k}^{(s)} \bigr) \\ =& \sum_{k=1}^{m-1}\frac{1}{k(m-k)} \bigl((m-k)B_{k}^{(r)}B_{m-k-1}^{(s-1)} + k B_{k-1}^{(r-1)} B_{m-k}^{(s)} \\ &{}+ k(m-k)B_{k-1}^{(r-1)}B_{m-k-1}^{(s-1)} \bigr). \end{aligned}$$

Then evidently we have

4.7 $$ \gamma_{m}(0)=\gamma_{m}(1)\quad\Leftrightarrow\quad \Lambda_{m} = 0 $$

and

4.8 $$ \int_{0}^{1}\gamma_{m}(x)\,dx = \frac{1}{m} \biggl(\Lambda_{m+1} - \frac{1}{m}B_{m}^{(r-1)} - \frac{1}{m}B_{m}^{(s-1)} \biggr). $$

We now would like to determine the Fourier coefficient $C_{n}^{(m)}$.

*Case* 1: $n\neq0$

4.9 $$\begin{aligned} C_{n}^{(m)} =& \int_{0}^{1}\gamma_{m}(x)e^{-2\pi i n x}\,dx \\ =&-\frac{1}{2\pi i n} \bigl[\gamma_{m}(x)e^{-2\pi i n x} \bigr]_{0}^{1} +\frac{1}{2\pi i n} \int_{0}^{1}\gamma'_{m}(x)e^{-2\pi i n x}\,dx \\ =&-\frac{1}{2\pi i n} \bigl(\gamma_{m}(1)-\gamma_{m}(0) \bigr) \\ &{} +\frac{1}{2\pi i n} \int_{0}^{1} \biggl((m-1)\gamma_{m-1}(x)+ \frac {1}{m-1}B_{m-1}^{(r)}(x)+ \frac{1}{m-1}B_{m-1}^{(s)}(x) \biggr)e^{-2\pi i n x}\,dx \\ =&\frac{m-1}{2\pi i n}C_{n}^{(m-1)}-\frac{1}{2\pi i n} \Lambda_{m} + \frac{1}{2\pi i n(m-1)} \int_{0}^{1}B_{m-1}^{(r)}(x)e^{-2\pi i n x}\,dx \\ &{}+ \frac{1}{2\pi i n(m-1)} \int_{0}^{1}B_{m-1}^{(s)}(x)e^{-2\pi i n x}\,dx \\ =&\frac{m-1}{2\pi i n} C_{n}^{(m-1)} - \frac{1}{2\pi i n} \Lambda_{m} - \frac{1}{2\pi i n(m-1)} \Phi_{m}^{(r)} \\ &{}- \frac{1}{2\pi i n(m-1)} \Phi_{m}^{(s)}, \end{aligned}$$

where

4.10 $$ \begin{aligned}[b] &\Phi_{m}^{(r)} = \sum_{k=1}^{m-1} \frac{(m-1)_{k}}{(2\pi i n)^{k}} B_{m-k-1}^{(r-1)}, \\ & \int_{0}^{1} B_{l}^{(r)}(x) e^{-2\pi i n x}\,dx = \textstyle\begin{cases} - \sum_{k=1}^{l} \frac{(l)_{k}}{(2\pi i n)^{k}} B_{l-k}^{(r-1)},&\mbox{for } n \neq0,\\ B_{l}^{(r-1)},&\mbox{for } n = 0. \end{cases}\displaystyle \end{aligned} $$

Thus we have shown that

4.11 $$ C_{n}^{(m)} = \frac{m-1}{2\pi i n} C_{n}^{(m-1)} - \frac{1}{2\pi i n} \Lambda_{m} - \frac{1}{2\pi i n(m-1)} \Phi_{m}^{(r)} - \frac{1}{2\pi i n(m-1)} \Phi_{m}^{(s)}, $$

from which, by induction on *m*, we can show that

4.12 $$ C_{n}^{(m)} = {-} \sum_{j=1}^{m-1} \frac{(m-1)_{j-1}}{(2\pi i n)^{j}} \Lambda_{m-j+1} - \sum_{j=1}^{m-1} \frac{(m-1)_{j-1}}{(2\pi i n)^{j} (m-j)} \bigl(\Phi _{m-j+1}^{(r)}+ \Phi_{m-j+1}^{(s)} \bigr). $$

Here we note that

4.13 $$\begin{aligned}& \sum_{j=1}^{m-1} \frac{(m-1)_{j-1}}{(2\pi i n)^{j}(m-j)}\Phi _{m-j+1}^{(r)} \\& \quad = \sum_{j=1}^{m-1} \frac{(m-1)_{j-1}}{(2\pi i n)^{j}(m-j)} \sum _{k=1}^{m-j}\frac{(m-j)_{k}}{(2\pi i n)^{k}}B_{m-j-k}^{(r-1)} \\& \quad = \sum_{j=1}^{m-1} \sum _{k=1}^{m-j} \frac{(m-1)_{j+k-1}}{(2\pi i n)^{j+k}(m-j)}B_{m-j-k}^{(r-1)} \\& \quad = \sum_{j=1}^{m-1} \frac{1}{m-j} \sum _{k=1}^{m-j} \frac {(m-1)_{j+k-1}}{(2\pi i n)^{j+k}}B_{m-j-k}^{(r-1)} \\& \quad = \sum_{j=1}^{m-1} \frac{1}{m-j} \sum _{k=j+1}^{m} \frac {(m-1)_{k-1}}{(2\pi i n)^{k}}B_{m-k}^{(r-1)} \\& \quad = \sum_{k=2}^{m} \frac{(m-1)_{k-1}}{(2\pi i n)^{k}}B_{m-k}^{(r-1)} \sum_{j=1}^{k-1} \frac{1}{m-j} \\& \quad = \sum_{k=1}^{m} \frac{(m-1)_{k-1}}{(2\pi i n)^{k}}B_{m-k}^{(r-1)} (H_{m-1} - H_{m-k} ) \\& \quad = \frac{1}{m} \sum_{k=1}^{m} \frac{(m)_{k}}{(2\pi i n)^{k}}B_{m-k}^{(r-1)} (H_{m-1} - H_{m-k} ). \end{aligned}$$

Finally, we get the following expression of $C_{n}^{(m)}$, for $n \neq0$:

4.14 $$ \begin{aligned} C_{n}^{(m)} & = - \frac{1}{m} \sum_{k=1}^{m} \frac{(m)_{k}}{(2\pi i n)^{k}} \bigl(\Lambda_{m-k+1} + (H_{m-1} - H_{m-k}) \bigl(B_{m-k}^{(r-1)} + B_{m-k}^{(s-1)} \bigr) \bigr). \end{aligned} $$

*Case* 2: $n=0$

4.15 $$ C_{0}^{(m)}= \int_{0}^{1}\gamma_{m}(x)\,dx= \frac{1}{m} \biggl(\Lambda _{m+1} - \frac{1}{m}B_{m}^{(r-1)} - \frac{1}{m}B_{m}^{(s-1)} \biggr). $$

$\gamma_{m}(\langle x\rangle )$, ($m \geq2$) is piecewise $C^{\infty}$. In addition, $\gamma_{m}(\langle x\rangle )$ is continuous for those integers $m \geq2$ with $\Lambda_{m} = 0$, and discontinuous with jump discontinuities at integers for those integer $m \geq2$ with $\Lambda_{m} \neq0$.

Assume first that $\Lambda_{m} = 0$, for an integer $m \geq2$. Then $\gamma_{m}(0)=\gamma_{m}(1)$. Hence $\gamma_{m}(\langle x\rangle )$ is piecewise $C^{\infty}$, and continuous. Thus the Fourier series of $\gamma _{m}(\langle x\rangle )$ converges uniformly to $\gamma_{m}(\langle x\rangle )$, and

4.16 $$\begin{aligned} \gamma_{m} \bigl(\langle x \rangle \bigr) =&\frac{1}{m} \biggl(\Lambda_{m+1} - \frac{1}{m}B_{m}^{(r-1)} - \frac{1}{m}B_{m}^{(s-1)} \biggr) \\ &{} + \frac{1}{m} \sum_{n=-\infty,n\neq0}^{\infty} \Biggl\{ -\sum_{k=1}^{m} \frac{(m)_{k}}{(2\pi i n)^{k}} \bigl(\Lambda_{m-k+1} + (H_{m-1} - H_{m-k}) \\ &{} \times \bigl(B_{m-k}^{(r-1)} + B_{m-k}^{(s-1)} \bigr) \bigr) \Biggr\} e^{2\pi i n x} \\ =& \frac{1}{m} \biggl(\Lambda_{m+1} - \frac{1}{m}B_{m}^{(r-1)} - \frac{1}{m}B_{m}^{(s-1)} \biggr) \\ &{} + \frac{1}{m} \sum_{k=1}^{m} \binom{m}{k} \bigl\{ \Lambda_{m-k+1} + (H_{m-1}-H_{m-k}) \bigl(B_{m-k}^{(r-1)} + B_{m-k}^{(s-1)} \bigr) \bigr\} \\ &{} \times \Biggl(-k!\sum_{n=-\infty,n\neq0}^{\infty} \frac{e^{2\pi i n x}}{(2\pi i n)^{k}} \Biggr) \\ =& \frac{1}{m} \biggl(\Lambda_{m+1} - \frac{1}{m}B_{m}^{(r-1)} - \frac{1}{m}B_{m}^{(s-1)} \biggr) \\ &{} + \frac{1}{m} \sum_{k=2}^{m} \binom{m}{k} \bigl\{ \Lambda_{m-k+1} + (H_{m-1}-H_{m-k}) \bigl(B_{m-k}^{(r-1)} + B_{m-k}^{(s-1)} \bigr) \bigr\} B_{k} \bigl(\langle x\rangle \bigr) \\ &{} + \Lambda_{m} \times \textstyle\begin{cases} B_{1}(\langle x\rangle ),&\mbox{for } x\notin\mathbb{Z},\\ 0,&\mbox{for } x\in\mathbb{Z} \end{cases}\displaystyle \\ =& \frac{1}{m} \sum_{k=0,k\neq1}^{m} \binom{m}{k} \bigl\{ \Lambda _{m-k+1} + (H_{m-1}-H_{m-k}) \bigl(B_{m-k}^{(r-1)} + B_{m-k}^{(s-1)} \bigr) \bigr\} B_{k} \bigl(\langle x\rangle \bigr) \\ &{} + \Lambda_{m} \times \textstyle\begin{cases} B_{1}(\langle x\rangle ),&\mbox{for } x\notin\mathbb{Z},\\ 0,&\mbox{for } x\in\mathbb{Z}. \end{cases}\displaystyle \end{aligned}$$

Now, we are ready to state our first result.

### Theorem 4.1

*For each integer* $l \geq2$, *let*

4.17 $$ \begin{aligned}[b] \Lambda_{l} ={}& \sum _{k=1}^{l-1}\frac{1}{k(l-k)} \bigl((l-k)B_{k}^{(r)}B_{l-k-1}^{(s-1)} + k B_{k-1}^{(r-1)} B_{l-k}^{(s)} \\ &{}+ k(l-k)B_{k-1}^{(r-1)}B_{l-k-1}^{(s-1)} \bigr), \end{aligned} $$

*with* $\Lambda_{1} = 0$. *Assume that* $\Lambda_{m} = 0$, *for an integer* $m \geq2$. *Then we have the following*.

(a) $\sum_{k=1}^{m-1}\frac{1}{k(m-k)} B_{k}^{(r)}(\langle x\rangle ) B_{m-k}^{(s)}(\langle x\rangle )$ *has the Fourier expansion*

4.18 $$ \begin{aligned}[b] & \sum_{k=1}^{m-1} \frac{1}{k(m-k)} B_{k}^{(r)} \bigl(\langle x\rangle \bigr) B_{m-k}^{(s)} \bigl(\langle x\rangle \bigr) \\ &\quad =\frac{1}{m} \biggl(\Lambda_{m+1} - \frac{1}{m}B_{m}^{(r-1)} - \frac{1}{m}B_{m}^{(s-1)} \biggr) \\ &\qquad{} + \frac{1}{m} \sum_{n=-\infty,n\neq0}^{\infty} \Biggl\{ -\sum_{k=1}^{m} \frac{(m)_{k}}{(2\pi i n)^{k}} \bigl(\Lambda_{m-k+1} + (H_{m-1} - H_{m-k}) \\ &\qquad{} \times \bigl(B_{m-k}^{(r-1)} + B_{m-k}^{(s-1)} \bigr) \bigr) \Biggr\} e^{2\pi i n x}, \end{aligned} $$

*for all* $x \in\mathbb{R}$, *where the convergence is uniform*.

4.19 $$ \begin{aligned}[b] &\mathrm{(b)}\quad \sum _{k=1}^{m-1} \frac{1}{k(m-k)} B_{k}^{(r)} \bigl(\langle x\rangle \bigr) B_{m-k}^{(s)} \bigl(\langle x \rangle \bigr) \\ &\phantom{\mathrm{(b)}\quad} \quad = \frac{1}{m} \sum_{k=0,k\neq1}^{m} \binom{m}{k} \bigl\{ \Lambda _{m-k+1} + (H_{m-1}-H_{m-k}) \bigl(B_{m-k}^{(r-1)} + B_{m-k}^{(s-1)} \bigr) \bigr\} B_{k} \bigl(\langle x\rangle \bigr) \end{aligned} $$

*for all* $x \in\mathbb{R}$.

Assume next that $\Lambda_{m} \neq0$, for an integers $m \geq2$. Then $\gamma_{m}(0) \neq\gamma_{m}(1)$. Hence $\gamma_{m}(\langle x\rangle )$ is piecewise $C^{\infty}$, and discontinuous with jump discontinuities at integers. Thus the Fourier series of $\gamma_{m}(\langle x\rangle )$ converges pointwise to $\gamma_{m}(\langle x\rangle )$, for $x\notin\mathbb{Z}$, and converges to

4.20 $$ \frac{1}{2} \bigl(\gamma_{m}(0)+ \gamma_{m}(1) \bigr) = \gamma_{m}(0) + \frac {1}{2} \Lambda_{m}, $$

for $x \in\mathbb{Z}$.

Now, we can state our second result.

### Theorem 4.2

*For each integer* $l \geq2$, *let*

4.21 $$ \begin{aligned}[b] \Lambda_{l} ={}& \sum _{k=1}^{l-1}\frac{1}{k(l-k)} \bigl((l-k)B_{k}^{(r)}B_{l-k-1}^{(s-1)} + k B_{k-1}^{(r-1)} B_{l-k}^{(s)} \\ &{}+ k(l-k)B_{k-1}^{(r-1)}B_{l-k-1}^{(s-1)} \bigr), \end{aligned} $$

*with* $\Lambda_{1} = 0$. *Assume that* $\Lambda_{m} \neq0$, *for an integer* $m \geq2$. *Then we have the following*:

4.22 $$\begin{aligned} & \begin{aligned}[b] &\mathrm{(a)}\quad \frac{1}{m} \biggl( \Lambda_{m+1} - \frac{1}{m}B_{m}^{(r-1)} - \frac{1}{m}B_{m}^{(s-1)} \biggr) \\ &\phantom{\mathrm{(b)}\quad}\qquad{} + \frac{1}{m} \sum_{n=-\infty,n\neq0}^{\infty} \Biggl\{ -\sum_{k=1}^{m} \frac{(m)_{k}}{(2\pi i n)^{k}} \bigl(\Lambda_{m-k+1} + (H_{m-1} - H_{m-k}) \\ &\phantom{\mathrm{(b)}\quad}\qquad{} {} \times \bigl(B_{m-k}^{(r-1)} + B_{m-k}^{(s-1)} \bigr) \bigr) \Biggr\} e^{2\pi i n x} \\ &\phantom{\mathrm{(b)}\quad}{}\quad = \textstyle\begin{cases} \sum_{k=1}^{m-1}\frac{1}{k(m-k)} B_{k}^{(r)}(\langle x\rangle ) B_{m-k}^{(s)}(\langle x\rangle ), &\textit{for } x \notin\mathbb{Z},\\ \sum_{k=1}^{m-1}\frac{1}{k(m-k)} B_{k}^{(r)}B_{m-k}^{(s)} + \frac {1}{2}\Lambda_{m}, &\textit{for } x \in\mathbb{Z}. \end{cases}\displaystyle \end{aligned} \end{aligned}$$

4.23 $$\begin{aligned} & \begin{aligned} & \mathrm{(b)}\quad \frac{1}{m} \sum _{k=0}^{m}\binom{m}{k} \bigl\{ \Lambda _{m-k+1} + (H_{m-1}-H_{m-k}) \bigl(B_{m-k}^{(r-1)} + B_{m-k}^{(s-1)} \bigr) \bigr\} B_{k} \bigl(\langle x \rangle \bigr) \\ &\phantom{\mathrm{(b)}\quad}\quad = \sum_{k=1}^{m-1} \frac{1}{k(m-k)} B_{k}^{(r)} \bigl(\langle x\rangle \bigr) B_{m-k}^{(s)} \bigl(\langle x\rangle \bigr), \quad\textit{for } x\notin\mathbb{Z}; \\ &\phantom{\mathrm{(b)}\quad} \frac{1}{m} \sum_{k=0,k\neq1}^{m} \binom{m}{k} \bigl\{ \Lambda _{m-k+1} + (H_{m-1}-H_{m-k}) \bigl(B_{m-k}^{(r-1)} + B_{m-k}^{(s-1)} \bigr) \bigr\} B_{k} \bigl(\langle x\rangle \bigr) \\ &\phantom{\mathrm{(b)}\quad} \quad = \sum_{k=1}^{m-1} \frac{1}{k(m-k)} B_{k}^{(r)}B_{m-k}^{(s)}+ \frac {1}{2} \Lambda_{m},\quad\textit{for } x\in\mathbb{Z}. \end{aligned} \end{aligned}$$

## Results and discussion

It is shown in a previous work that Faber-Pandharipande-Zagier’s and Miki’s identities can be derived from a polynomial identity, which in turn follows from the Fourier series expansion of sums of products of Bernoulli functions. Motivated by and generalizing this, we consider three types of functions given by sums of products of higher-order Bernoulli functions and we obtain some new identities arising from Fourier series expansions associated with sums of products of higher-order Bernoulli functions. Moreover, we will express each of them in terms of Bernoulli functions. The Fourier series expansion of the sums of products of higher-order Bernoulli functions are useful in computing the special values of the zeta and multiple zeta function. It is expected that the Fourier series of the sums of products of higher-order Bernoulli functions will find some applications in connection with a certain zeta function and the higher-order Bernoulli numbers.

## Conclusion

In this paper, we considered the Fourier series expansion of the sums of products of higher-order Bernoulli functions which are obtained by extending by periodicity of period the Bernoulli polynomials on $[0, 1)$. The Fourier series are explicitly determined.
